# circNFIC suppresses breast cancer progression by sponging miR-658

**DOI:** 10.7150/jca.38830

**Published:** 2020-04-27

**Authors:** Gaosheng Xu, Dongmei Ye, Qiang Zhao, Rongfang He, Wei Ma, Yuxuan Li, Shujie Tang, Zhiwei Zhou, Xing Li, Zhiwei Zhang

**Affiliations:** 1 Yueyang Maternal and Child Health Hospital, Yueyang; Innovative Practice Base for Postgraduate Training of Basic Medicine and Clinical Collaboration, University of South China and Yueyang Maternal and Child Health Hospital, Yueyang 414000, Hunan Province, China.; 2 Key Laboratory of Cancer Cellular and Molecular Pathology in Hunan Province, Cancer Research Institute of Hengyang Medical College, University of South China, Hengyang, 421001, Hunan Province, China;; 3 Department of Pathology, the First Affiliated Hospital of University of South China, Hengyang, 421001, Hunan Province, China;; 4 Clinical Medicine Undergraduate Program, Medical College, University of South China, Hengyang, 421001, Hunan Province, China.; 5 Sun Yat-sen University Cancer Center, State Key Laboratory of Oncology in South China, Collaborative Innovation Center for Cancer Medicine, 651 Dongfeng East Road, Guangzhou, 510060, China; Gaosheng Xu and Dongmei Ye contributed equally to this work.

**Keywords:** breast cancer, circRNA, competitive endogenous RNA, proliferation, migration

## Abstract

**Background**: Circular RNAs (circRNAs) have been reported to play important roles in cancer progression. However, the potential involvement of circRNAs in breast cancer metastasis to the lung remains unclear.

**Methods**: High-throughput circular RNA microarray assays of primary breast cancer tissues and lung metastatic tissues were performed. Reactome pathway analysis and GO analysis of the linear mRNA transcripts corresponding to the circRNAs were conducted. The expression of the top downregulated circRNA was confirmed by qRT-PCR in breast cancer cell lines. Kaplan-Meier survival analysis was conducted to analyze the clinical significance of the selected circRNA in breast cancer. A series of *in vitro* and *in vivo* experiments, including cell proliferation and migration, was performed to explore the functions of the selected circRNA in breast cancer progression. We further investigated the regulatory effect of the selected circRNA on a miRNA and its target genes to explore the potential mechanisms.

**Results**: We found that circNFIC (hsa_circ_0002018) was the most downregulated circRNA in lung metastatic tissues. Kaplan-Meier survival analysis revealed that low levels of circNFIC were related to poor outcome of breast cancer. Further experiments revealed that overexpressing circNFIC suppressed breast cancer cell proliferation and migration to the lung. A mechanistic study showed that circNFIC acted as a sponge for miR-658 and competed for binding to miR-658 with UPK1A, leading to increased expression of UPK1A.

**Conclusion**: Our study highlighted the regulatory function of the circNFIC/miR-658/UPK1A pathway in breast cancer progression, which could be a potential therapeutic target for breast cancer.

## Introduction

Cancer metastasis is responsible for more than 90% of cancer-related deaths 1. In breast cancer, many noncoding RNAs are reported to be involved in lung metastasis. miRNA microarray profiling revealed that miR-374a was most strongly upregulated in lung metastatic derivatives of breast cancer cell lines and that miR-374a levels correlated with metastatic recurrence in breast cancer patients 2. lncRNA MALAT1 was identified as a metastasis-suppressing lncRNA in breast cancer that was inversely correlated with breast cancer progression and metastatic ability 3. However, the involvement of circular RNAs (circRNAs) in breast cancer lung metastasis remains unknown.

circRNAs are considered important regulators and biomarkers of cancer progression. Furthermore, circRNAs have been shown to function as miRNA sponges or competitive endogenous RNAs (ceRNAs) 4. In triple negative breast cancer, circAGFG1 promoted cell proliferation, mobility and invasion by acting as a ceRNA of miR-195 to relieve the repression of cyclin E1 expression 5. Moreover, circ_0006528 promoted breast cancer cell proliferation, invasion and migration by sponging miR-7 to promote the expression of Raf1 and activate the MAPK/ERK pathway 6. All these studies suggest that circRNAs are potential therapeutic targets and prognostic predictors for breast cancer and deserve deeper exploration.

In this study, we performed high-throughput circular RNA microarray assays of primary breast cancer tissues and lung metastatic tissues to explore the potential involvement of circRNAs in breast cancer lung metastasis. Kaplan-Meier survival analysis and a series of experiments were conducted to analyze the clinical significance and functions of the selected circRNA in breast cancer. The regulatory effects and potential mechanisms of the selected circRNA on a miRNA and its target genes were further investigated.

## Material and Methods

### Patient samples

Primary breast cancer tissues and lung metastatic tissues were collected from Nanhua Affiliated Hospital and subjected to circRNA microarray analysis. Breast cancer tissues from 150 patients were collected from Nanhua Affiliated Hospital and subjected to qRT-PCR. Patients who underwent curative surgical treatment (mastectomy or breast-conserving surgery with axillary evaluation) and did not receive any chemotherapy or radiation therapy beforehand were recruited. The exclusion criteria included male patients, inflammatory breast carcinomas, bilateral carcinomas, and history of any malignant tumor. The characteristics of the 150 breast cancer patients are summarized in Table 1.

### Microarray analysis

CircRNA microarray analysis was performed with CapitalBio Technology Human CircRNA Array v2. Data were analyzed with GeneSpring software V13.0 (Agilent). The result was log2 transformed and median centered by genes with CLUSTER 3.0 software and analyzed by hierarchical clustering with average linkage.

### Cell culture and transfection

Breast cell lines were purchased from American Type Culture Collection (ATCC, USA). Cells were cultured according to the supplier's instructions. DNA fingerprinting was conducted to verify cell authenticity.

Cells were transfected using Lipofectamine 2000 (Invitrogen, USA). A vector overexpressing circNFIC, miR-658 mimics and inhibitors were purchased from GeneCopoeia.

### Quantitative real-time PCR (qRT-PCR)

RNA was isolated by TRIzol (Invitrogen). Nuclear and cytoplasmic fractions were isolated by NE-PER Nuclear and Cytoplasmic Extraction Reagents (Thermo Scientific, USA). qRT-PCR was performed with SYBR Premix Ex Taq (Takara Bio Inc., China) and All-in-One™miRNA qRT-PCR Detection Kit (GeneCopoeia, USA). Primers were synthesized by Invitrogen (Supplementary Table 1).

### Cell counting kit-8 (CCK-8) assay

Cell proliferation was assessed by CCK-8 assay (Dojindo Laboratories, Japan). Cells (1×10^3^) were seeded into 96-well plates, and CCK-8 solution (10 μl) was added 48 h after transfection. After 2 h of incubation at 37 °C, the absorbance at 450 nM was measured in a microtiter plate reader (Bio-Tek EPOCH2, USA).

### Colony formation assay

Cells were seeded in 6-well plates at a density of 1×10^3^ cells/well and incubated at 37 °C for 2 weeks. Colonies were fixed in methanol, stained with 0.1% crystal violet, imaged and counted.

### Mouse xenograft model

Cells (2×10^6^) were subcutaneously injected into the dorsal flanks of 4-week-old female BALB/c nude mice (three mice per group), and the mice were then treated with an intratumoral injection (40 μL NC or circNFIC) every 4 days. Xenografts were excised under anesthesia after 4 weeks, and the tumor weights were measured.

For lung metastasis studies, 1 × 10^5^ cells were injected into the tail veins of mice (three mice per group). After 8 weeks, the lungs were excised under anesthesia, and the numbers of macroscopically visible lung metastatic nodules were counted and validated by assessment of hematoxylin and eosin (HE)-stained sections by microscopy.

### Luciferase reporter assay

Cells (5×10^3^) were seeded and cotransfected with the corresponding plasmids and miR-658 mimics using Lipofectamine 2000. After 48 h of incubation, luciferase activities were quantified with a Dual-Luciferase Reporter Assay System (Promega, USA).

### RNA immunoprecipitation (RIP) assay

Cells were cotransfected with MS2bs-circNFIC, MS2bs-circNFIC-mt or blank control MS2bs-Rluc together with MS2bp-GFP using Lipofectamine 2000. After 48 h, RIP was performed with a GFP antibody (Roche, USA) and a Magna RIP RNA-Binding Protein Immunoprecipitation Kit (Millipore, USA). The RNA complexes were then purified, and the level of miR-660 was quantified.

For the RIP assay for Ago2, 48 h after transfection, RIP was performed with an anti-Ago2 antibody (Millipore), and the levels of circNFIC, UPK1A and miR-658 were measured.

### Statistical analysis

Comparisons between groups were analyzed using t tests. Kaplan-Meier plots and log-rank tests were used for the survival analysis. Unless otherwise indicated, the data are presented as the mean ± S.D. of three independent experiments. *P* < 0.05 was considered statistically significant. The statistical analysis was performed using SPSS 19.0 software.

## Results

### circNFIC is downregulated in breast cancer and correlated with poor outcome

**Figure 1:**
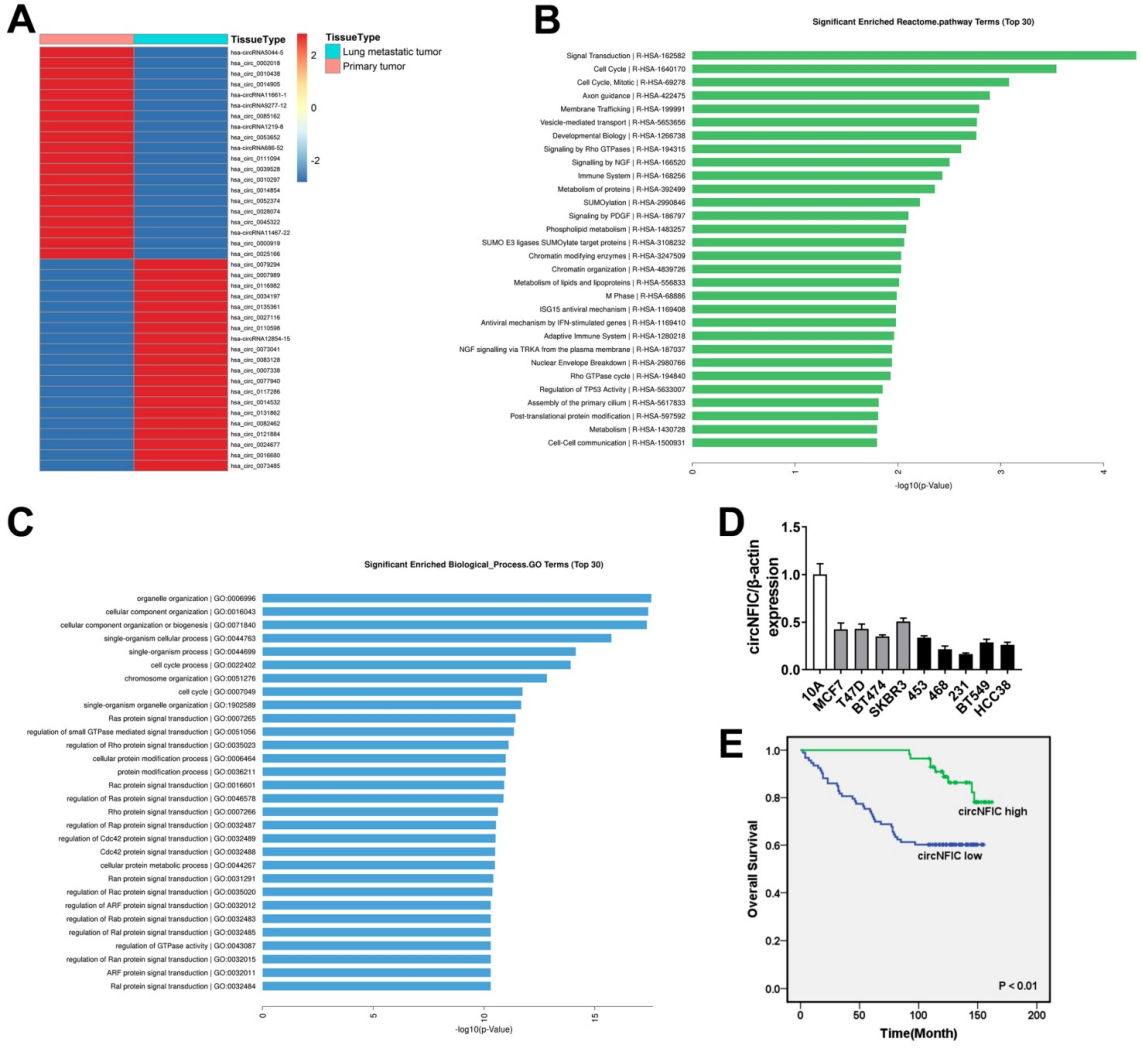
** circNFIC is downregulated in breast cancer and is correlated with poor outcome A.** Hierarchical cluster analysis showing the top 20 upregulated and downregulated circRNAs in lung metastatic tissues compared with primary breast cancer tissues: red, upregulated; blue, downregulated. **B.** Reactome pathway analysis was performed. **C.** GO analysis was performed. **D.** The expression of circNFIC in breast cancer cell lines. **E.** OS curves for 150 breast cancer patients with high or low circNFIC expression.

To investigate the potential involvement of circRNAs in breast cancer metastasis to the lung, we performed high-throughput circular RNA microarray assays of primary breast cancer tissues and lung metastatic tissues. Hierarchical clustering showed the top 20 upregulated and downregulated circRNAs based on fold change ≥ 2 (Figure 1A). Reactome pathway analysis of the linear mRNA transcripts corresponding to the circRNAs revealed that the corresponding linear mRNAs were related to cell cycle pathways (Figure 1B). Moreover, GO analysis showed that the corresponding linear mRNAs were related to cell cycle progression, indicating their potential involvement in cancer progression (Figure 1C). Among the top 20 downregulated circRNAs, hsa_circ_0002018 decreased the most in lung metastatic tissues; therefore, we decided to study this circRNA. Hsa_circ_0002018 (chr19: 3449011-3452664) was predicted to derive from nuclear factor I C (NFIC) by the human reference genome (GRCh37/hg19). Thus, we named hsa_circ_0002018 "circNFIC".

We measured circNFIC expression in breast cancer cell lines and found that circNFIC was downregulated in breast cancer cell lines, especially in MDA-MB-468 and MDA-MB-231 cells (Figure 1D). Therefore, we used these cell lines in the following study. To explore the clinical significance of circNFIC in breast cancer, we performed survival analysis on 150 breast cancer patients. The characteristics of these patients are summarized in Table 1. Patients with circNFIC expression equal to or greater than the average were assigned to the “circNFIC high” group. We explored the potential clinicopathological implications of circNFIC in breast cancer. We found that low levels of circNFIC were related to shorter overall survival and lymph node metastasis (*P* = 0.002 and 0.021, respectively). Kaplan-Meier survival analysis revealed that low levels of circNFIC were related to poor outcome of breast cancer, indicating the vital role circNFIC plays in breast cancer progression (Figure 1E).

### Overexpressing circNFIC suppresses the proliferation and migration of breast cancer cells

**Figure 2:**
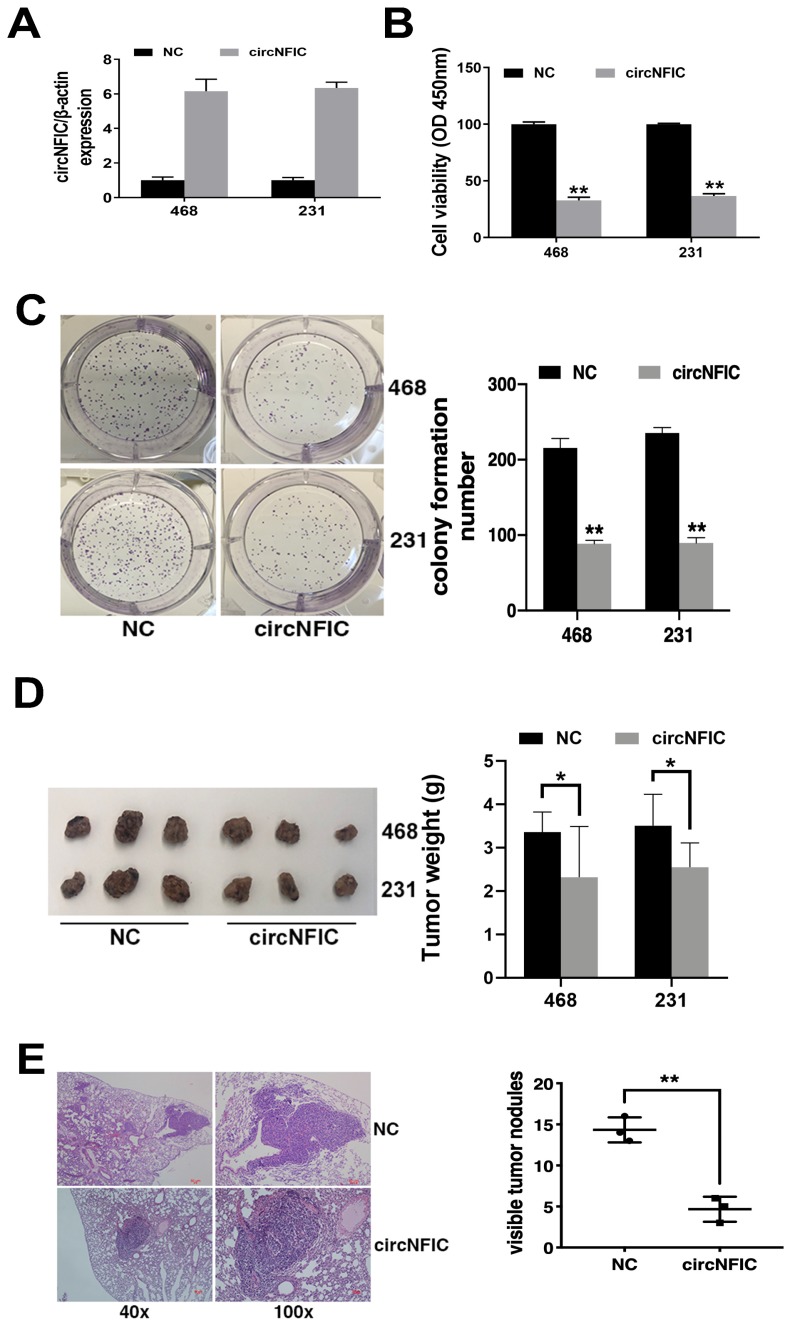
** Overexpressing circNFIC suppresses the proliferation and migration of breast cancer cells A.** circNFIC was successfully overexpressed in breast cancer cell lines. **B.** CCK-8 assay was performed to assess cell proliferation. **C.** Colony formation assay was performed to assess cell colony-forming ability (left), and the colony formation number was quantified by ImageJ (right). **D.** Representative images of mouse xenograft tumors (left) and tumor weights are summarized (right).** E.** Representative images of lung metastatic nodules in HE-stained sections (left). The number of metastatic nodules was quantified (right). *P < 0.05, **P < 0.01

To explore the function of circNFIC in breast cancer, we overexpressed circNFIC in breast cancer cell lines (Figure 2A). The CCK-8 assay showed that circNFIC overexpression significantly suppressed cell proliferation (Figure 2B). circNFIC overexpression also reduced the colony formation ability of breast cancer cells (Figure 2C).

To further explore the function of circNFIC *in vivo*, mouse xenograft models were established. The results showed that circNFIC overexpression significantly decreased tumor growth (Figure 2D) and lung metastasis (Figure 2E), indicating that circNFIC overexpression suppresses cell proliferation and migration in breast cancer.

### circNFIC acts as a sponge for miR-658

**Figure 3:**
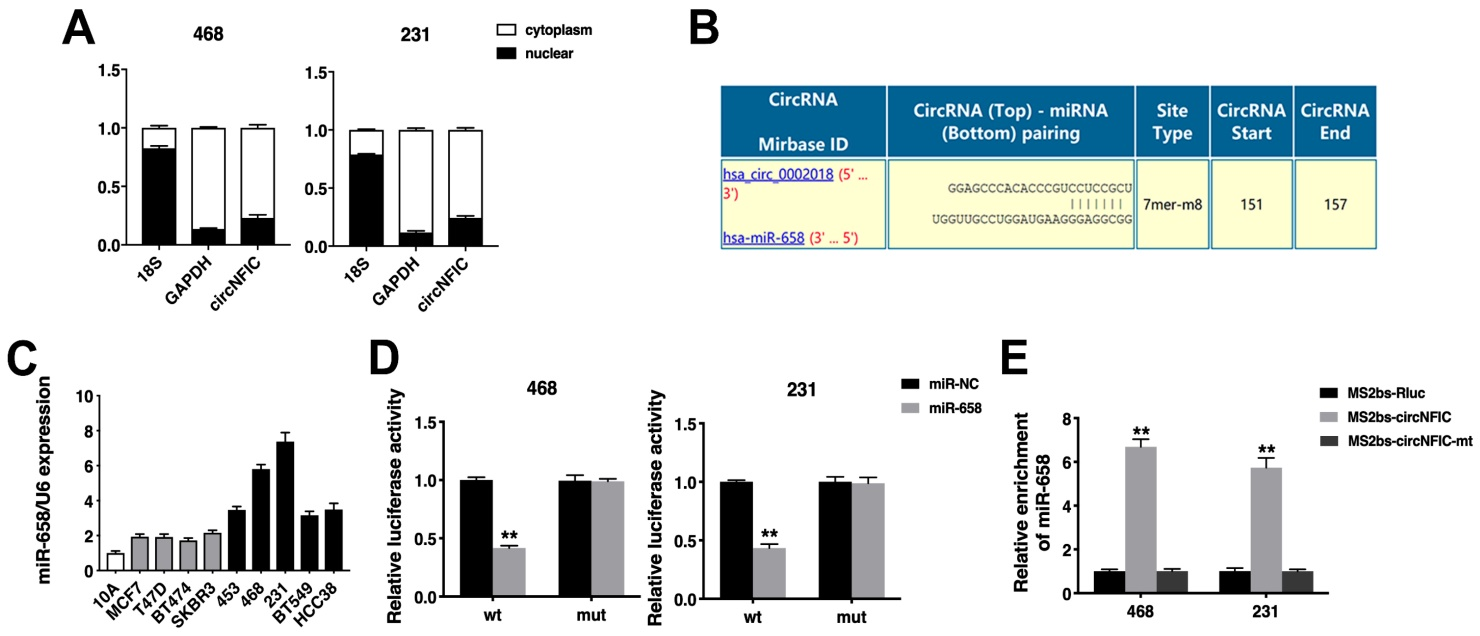
** circNFIC acts as a sponge for miR-658 A.** The levels of nuclear control transcript (18S), cytoplasmic control transcript (GAPDH) and circNFIC were assessed in nuclear and cytoplasmic fractions. **B.** The predicted binding sites of miR-658 within the circNFIC sequence. **C.** The expression of miR-658 in breast cancer cell lines. **D.** Luciferase assay of cells cotransfected with miR-658 mimics and wild-type or mutant luciferase reporter. **E.** MS2-based RIP assay in cells transfected with MS2bs-circNFIC, MS2bs-circNFIC-mt or control. **P < 0.01

Next, we detected the intracellular location of circNFIC and found that circNFIC predominantly localized in the cytoplasm, indicating that circNFIC might function as a miRNA sponge (Figure 3A). Therefore, we used Circular RNA Interactome (https://circinteractome.nia.nih.gov/index.html) to predict the potential circRNA/miRNA interactions, and binding sites for miR-658 were found within the circNFIC sequence (Figure 3B). We then detected the expression of miR-658 in breast cancer cell lines and found that miR-658 was upregulated (Figure 3C). A luciferase reporter assay showed that the luciferase activity was reduced after cotransfection of the wild-type luciferase reporter and miR-658 mimics, while the mutated luciferase reporter exerted no such effect (Figure 3D). To confirm the direct binding of circNFIC and miR-658, a RIP assay was performed. The results showed that miR-658 was predominantly enriched in RNAs retrieved from MS2bs-circNFIC, indicating that circNFIC directly interacts with miR-658 and could act as a sponge for miR-658 (Figure 3E).

### circNFIC acts as a ceRNA to regulate UPK1A

**Figure 4:**
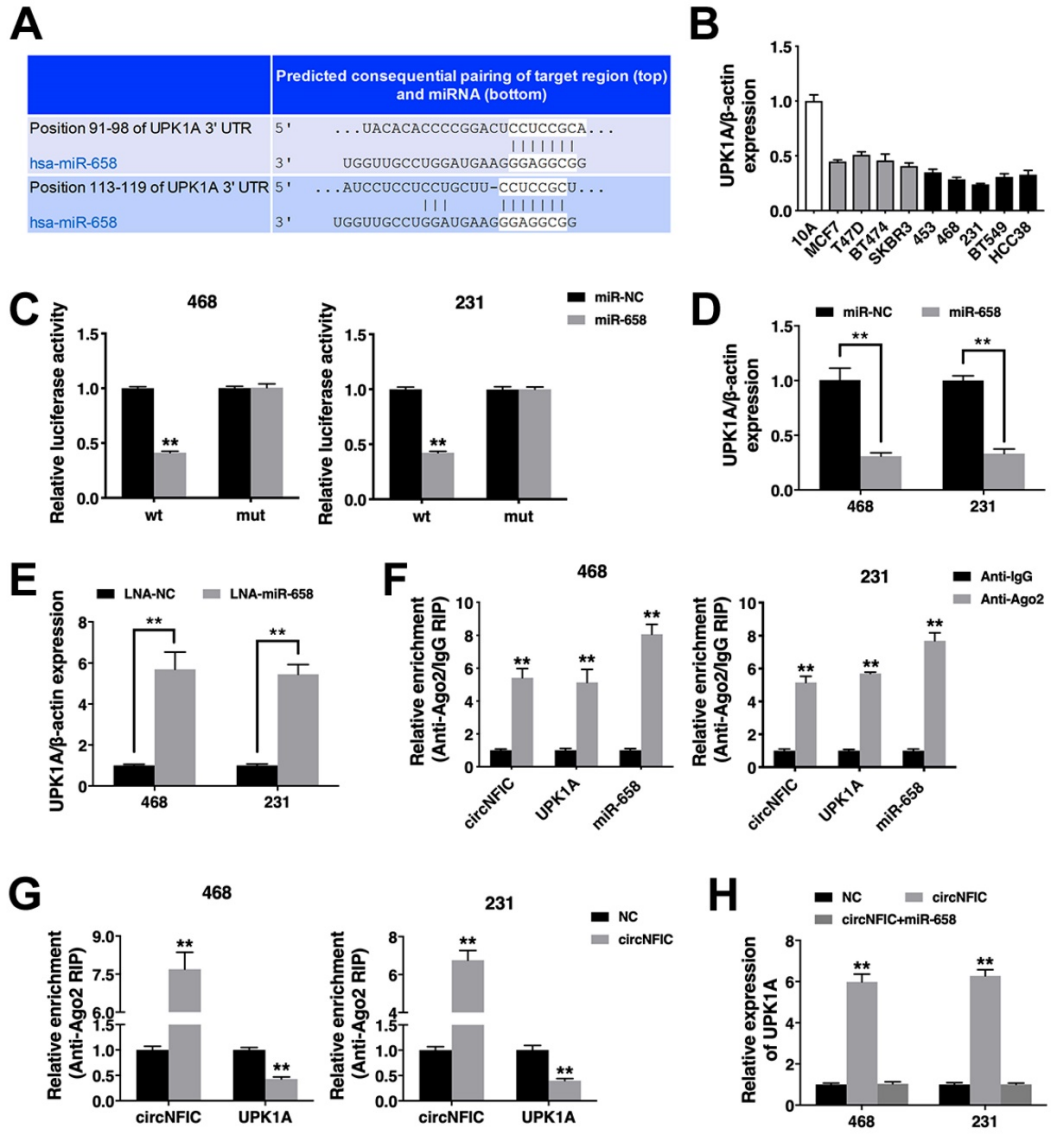
** circNFIC acts as a ceRNA to regulate UPK1A A.** The predicted binding sites of miR-658 within the UPK1A 3'UTR. **B.** The expression of UPK1A in breast cancer cell lines. **C.** Cells were transfected, and a luciferase assay was performed. **D.** Cells were transfected, and the expression of UPK1A was detected. **E.** The expression of UPK1A was detected. **F.** RIP assay showing the enrichment of circNFIC, UPK1A and miR-658 in the Ago2 fraction relative to the IgG fraction. **G.** Cells were transfected, and a RIP assay on Ago2 was performed. **H.** Cells were transfected, and the expression of UPK1A was detected. **P < 0.01

To validate whether circNFIC sponges miR-658 and restores the expression of its target, we searched TargetScan for potential target genes of miR-658, and Uroplakin 1A (UPK1A) was predicted (Figure 4A). Thus, we explored UPK1A expression in breast cancer cell lines and found that UPK1A was downregulated (Figure 4B). A luciferase reporter assay revealed decreased luciferase activity after cotransfection of miR-658 mimics and the wild-type luciferase reporter, while the mutated luciferase reporter exerted no such effect (Figure 4C). Furthermore, miR-658 suppressed UPK1A expression, while the miR-658 inhibitor increased UPK1A expression, indicating that UPK1A is a target gene of miR-658 and could be regulated by miR-658 (Figure 4D & 4E).

Next, a RIP assay on Ago2 showed that circNFIC, UPK1A and miR-658 were mainly enriched in Ago2-bound transcripts, indicating that circNFIC and UPK1A are recruited to an Ago2-related RISC where they interact with miR-658 (Figure 4F). Additionally, overexpressing circNFIC increased the enrichment of circNFIC on Ago2 but decreased the enrichment of UPK1A on Ago2, indicating that circNFIC could function as a ceRNA and compete with UPK1A to bind the miRNA (Figure 4G). Furthermore, circNFIC overexpression led to increased UPK1A expression, while miR-658 reversed this increase, indicating that circNFIC sponges miR-658 to regulate UPK1A expression (Figure 4H).

## Discussion

Metastasis accounts for approximately 90% of cancer patient deaths 7. Noncoding RNAs have been reported to be critical for the metastatic ability of cancer cells and therefore have been considered potential therapeutic targets 8. In breast cancer, ectopic miR-126 expression significantly suppressed the formation of lung metastases, while suppressing miR-126 significantly increased the ability of breast cancer cells to metastasize to the lung 9. For circRNAs, circANKS1B was found to be upregulated in triple-negative breast cancer and could promote breast cancer invasion and metastasis by inducing epithelial-to-mesenchymal transition 10, demonstrating that circRNAs could be therapeutic targets to better prevent breast cancer metastasis.

In this study, we performed high-throughput circular RNA microarray assays of primary breast cancer tissues and lung metastatic tissues and found that circNFIC (hsa_circ_0002018) was the most downregulated circRNA in lung metastatic tissues. In addition, circNFIC was confirmed to be downregulated in breast cancer cell lines. Moreover, low levels of circNFIC were related to poor outcome of breast cancer, indicating the vital role circNFIC plays in breast cancer progression. We further overexpressed circNFIC in breast cancer cell lines and found that circNFIC overexpression significantly suppressed cell proliferation and lung metastasis.

It has been reported that circRNAs could serve as ceRNAs to regulate cancer progression via competitive binding to shared miRNAs 11. In breast cancer, circIRAK3 sponged miR-3607 to regulate the expression of forkhead box C1 and further mediated breast cancer cell migration 12. circTADA2A-E6 suppressed breast cancer cell proliferation, migration, invasion and clonogenicity by acting as a miR-203a-3p sponge to restore the expression of SOCS3 13. Furthermore, hsa_circ_0072995 regulated breast cancer invasion and migration by sponging miR-30c-2-3p 14. Here, a mechanistic study revealed that circNFIC could also act as a sponge for miR-658 to regulate breast cancer progression.

miR-658 was found to be highly expressed in gastric cancer tissues and cells 15. Elevated circulating miR-658 was associated with tumor metastasis through activation of the PAX3-MET pathway 16. Here, we found that miR-658 was upregulated in breast cancer cell lines, and circNFIC could directly interact with miR-658 and act as a sponge for miR-658 to regulate the expression of UPK1A, a target gene of miR-658.

UPK1A has long been reported to be a tumor suppressor gene in multiple cancers and could be a useful marker for diagnosis and prognostic prediction. In esophageal squamous cell carcinoma, UPK1A was found to be downregulated, and UPK1A could inhibit cell proliferation, clonogenicity, cell motility and tumor formation 17. In gastric cancer, UPK1A was reported to be downregulated, and increased expression of UPK1A could inhibit cell migration and invasion 18. In colorectal cancer, UPK1A was also downregulated, and low expression of UPK1A was significantly associated with poorer survival 19. Here, we found that UPK1A was downregulated in breast cancer cell lines and that circNFIC could sponge miR-658 to regulate the expression of UPK1A and the progression of breast cancer.

Taken together, our study indicates that circNFIC is downregulated in breast cancer and is related to poor outcome. Overexpression of circNFIC inhibits cell proliferation and migration in breast cancer. Moreover, circNFIC functions as a ceRNA to regulate UPK1A expression by decoying miR-658. circNFIC can be used as a diagnostic biomarker and therapeutic target for breast cancer.

## Conclusions

circNFIC could be used as a promising prognostic biomarker for breast cancer patients, and therapeutic targeting of the circNFIC/miR-658/UPK1A network may be a potential strategy for the treatment of breast cancer.

## Supplementary Material

Supplementary table.

## Figures and Tables

**Table 1:** Clinicopathological variables and circNFIC expression in 150 breast cancer patients

Characteristics	Total (n=150)	circNFIC high (n=57)		circNFIC low (n=93)		*P* value
		No.	%	No.	%	
OS						0.002*
Present	104	48	46.2	56	53.8	
Absent	46	9	19.6	37	80.4	
Age (years)						0.515
<50	66	27	40.9	39	59.1	
≥50	84	30	35.7	54	64.3	
Tumor size (cm)**						0.473
≤2	34	11	32.4	23	67.6	
>2	115	45	39.1	70	60.9	
LNMET**						0.021*
Yes	91	28	30.8	63	69.2	
No	54	27	50.0	27	50.0	
TNM stage**						0.182
I-II	97	40	41.2	57	58.8	
III- IV	50	15	30.0	35	70.0	
ER status**						0.059
Positive	93	41	44.1	52	55.9	
Negative	50	14	28.0	36	72.0	
PR status**						0.324
Positive	81	34	42.0	47	58.0	
Negative	62	21	33.9	41	66.1	
HER-2 status**						0.689
Positive	41	17	41.5	24	58.5	
Negative	103	39	37.9	64	62.1	

* indicates statistical significance (*P* < 0.05). % indicates percentage within the row. ** The case number does not add up to the total in several categories because a few patients were excluded from those categories.
